# On-Site Monitoring of Postoperative Bile Leakage Using Bilirubin-Inducible Fluorescent Protein

**DOI:** 10.1007/s00268-020-05774-x

**Published:** 2020-09-09

**Authors:** Yoshiharu Kono, Takeaki Ishizawa, Norihiro Kokudo, Yugo Kuriki, Ryu J. Iwatate, Mako Kamiya, Yasuteru Urano, Akiko Kumagai, Hiroshi Kurokawa, Atsushi Miyawaki, Kiyoshi Hasegawa

**Affiliations:** 1 Hepatobiliary-Pancreatic Surgery Division, Department of Surgery, Graduate School of Medicine, The University of Tokyo, 7-3-1 Hongo, Bunkyo, Tokyo 113-8655 Japan; 2 Department of Gastroenterological Surgery, Cancer Institute Hospital, Japanese Foundation for Cancer Research, Tokyo, Japan; 3 Laboratory of Chemistry and Biology, Graduate School of Pharmaceutical Sciences, The University of Tokyo, Tokyo, Japan; 4 Laboratory of Chemical Biology and Molecular Imaging, Graduate School of Medicine, The University of Tokyo, Tokyo, Japan; 5 Japan Science and Technology Agency, PRESTO, Saitama, Japan; 6 Japan Agency for Medical Research and Development, CREST, Tokyo, Japan; 7 Laboratory for Cell Function Dynamics, RIKEN Centre for Brain Science, 2-1 Hirosawa, Wako, Saitama 351-0198 Japan

## Abstract

**Background:**

Bile leakage is the most common postoperative complication associated with hepatobiliary and pancreatic surgery. Until now, however, a rapid, accurate diagnostic method for monitoring intraoperative and postoperative bile leakage had not been established.

**Method:**

Bilirubin levels in drained abdominal fluids collected from 23 patients who had undergone hepatectomy (*n* = 22) or liver transplantation (*n* = 1) were measured using a microplate reader with excitation/emission wavelengths of 497/527 nm after applying 5 µM of UnaG to the samples. UnaG was also sprayed directly on hepatic raw surfaces in swine hepatectomy models to identify bile leaks by fluorescence imaging.

**Results:**

The bilirubin levels measured by UnaG fluorescence imaging showed favorable correlations with the results of the conventional light-absorptiometric methods (indirect bilirubin: rs = 0.939, *p* < 0.001; direct bilirubin: rs = 0.929, *p* < 0.001). Approximate time required for bilirubin measurements with UnaG was 15 min, whereas it took about 40 min with the conventional method at a hospital laboratory. Following administration of UnaG on hepatic surfaces, the fluorescence imaging identified bile leaks not only on the resected specimens but also in the abdominal cavity of the swine hepatectomy models.

**Conclusion:**

Fluorescence imaging techniques using UnaG may enable real-time identification of bile leaks during hepatectomy and on-site rapid diagnosis of bile leaks after surgery.

**Electronic supplementary material:**

The online version of this article (10.1007/s00268-020-05774-x) contains supplementary material, which is available to authorized users.

## Introduction

One of the major complications after hepatobiliary and pancreatic surgery has been postoperative bile leakage, the incidences of which are reportedly 3–12% after hepatectomy [1–8] and 2–10% after pancreatoduodenectomy [9–11]. While more and more liver surgeons have recently advocated a “no drain” policy for decreasing a risk of infection and postoperative hospital stay [5, 8, 12, 13], prophylactic abdominal drains are still used for the diagnosis and management of postoperative bile leak, especially in patients at high risk of complications after liver resection or transplantation [5, 14–16]. Even when no prophylactic drains are used during surgery, additional drainage tubes may be placed postoperatively to drain symptomatic intraabdominal fluid collections associated with bile leakage. Thus, it remains important to evaluate the presence and severity of bile leaks from hepatic raw surfaces and/or biliary anastomoses intraoperatively and postoperatively by studying the characteristics of the leaked abdominal fluids.

During the management of patients with abdominal drains after hepatobiliary and pancreatic surgery, bilirubin levels are usually measured in the drained abdominal fluids to directly monitor the patient for postoperative bile leakage. A treatment policy has established the acceptable upper limit of the total bilirubin (TB) level in the fluid at 3 mg/dL [4, 6] or three times the serum TB level [16]. When the TB level decreases to values below either of the limits, the clinicians may decide to remove the drainage tube. It requires time and additional work for the medical staff to collect and send abdominal fluid samples to hospital laboratories and then for the laboratory personnel to measure the fluids’ bilirubin levels after removing impurities. Although most clinical laboratories are currently equipped with automatic biochemical analyzers for blood samples, the available light-absorptiometric methods for bilirubin measurement are insensitive, indirect, and have not changed since 1916 [17]. Thus, an efficient, accurate method is essential for bedside measurement of bilirubin levels in drainage fluids, which would greatly improve postoperative management.

UnaG is a unique ligand-inducible fluorescent protein cloned from eel muscle [18]. It binds unconjugated bilirubin non-covalently as a fluorogenic ligand with high specificity and affinity (*K*_d_ = 98 pM). The resulting UnaG–bilirubin complex (holoUnaG) emits green fluorescence ($\lambda_{\max }$ = 527 nm, $\Phi_{f}$ = 0.51) under blue-light illumination. The use of ligand-free UnaG (apoUnaG) allowed the development of a direct fluorometric assay with clinical application that is potentially superior in sensitivity, speed, reproducibility, economy, and simplicity to the currently used light-absorptiometric methods [18, 19]. In addition, UnaG has a potential to be used for highly sensitive detection of bile juice by intraoperative fluorescence imaging.

In the present study, we first applied apoUnaG to hepatobiliary surgery for monitoring bilirubin levels in drained abdominal fluids collected from human patients, aiming to establish bedside quantitative diagnosis of postoperative bile leak. Second, we used apoUnaG to directly visualize bile leakage on hepatic raw surfaces using swine hepatectomy models.

## Methods

### Ethical considerations

This study was conducted with the approval of the Institutional Ethics Review Board of The University of Tokyo and was registered in the UMIN Clinical Trials Registry (Registration Number: UMIN000003654; https://www.umin.ac.jp/ctr/index.htm). Informed consent was obtained from all patients. The animal experiments complied with the ARRIVE guidelines, and they were carried out in strict accordance with the recommendations in the Guide for the Care and Use of Laboratory Animals from the National Institutes of Health. The protocol for the animal experiments was approved by the institutional committee on the Ethics of Animal Experiments. We performed a laparoscopic limited resection of the liver on a 4-month male pig (60 kg liveweight) to create a swine hepatectomy model. All surgery was performed under isoflurane anesthesia, and all possible efforts were made to minimize suffering.

### Sample collection and bilirubin measurements in postoperative abdominal drainage fluids using conventional techniques

Subjects consisted of 22 patients who underwent hepatectomy and 1 recipient who underwent living-donor liver transplantation at The University of Tokyo between June and December 2014. During the surgical operations, hepatic parenchyma was dissected using the clamp-crushing method with the use of a Cavitron ultrasonic surgical aspirator and/or vessel sealing system under intermittent inflow occlusion. Prophylactic abdominal drains were routinely placed and connected to fluid collection bags.

After surgery, drained fluid and blood samples were obtained daily on postoperative days 1–7. These samples were submitted to the in-hospital biochemical testing room. In the hospital laboratory, these samples were first centrifuged for 5 min after 10 min of remaining still. They were then placed in an automated biochemical analyzer to calculate the TB and direct bilirubin levels (using the conventional enzymatic method—i.e., absorption spectrophotometry following a reaction with bilirubin oxidase—using commercially available reagents (NESCAUTO®VL T-BIL and D-BIL; Alfresa-Pharma, Osaka, Japan). The indirect bilirubin (IDB) level was calculated by subtracting the direct bilirubin from the TB. It took approximately 40 min from sample preparation to completion of calculating the bilirubin levels. The remaining samples were frozen and stored at − 80 °C until direct measurement of the IDB level using apoUnaG.

### Estimating bilirubin levels in postoperative abdominal drainage fluids using apoUnaG

The recombinant apoUnaG protein was expressed in *Escherichia coli* (JM109[DE3]) and purified using Ni^2+^ chromatography, as described previously [18].

The reaction solution of the fluorometric method contained phosphate-buffered saline with 5 µM apoUnaG. After thawing the abdominal fluid samples, 1 µl of drainage fluid was mixed with 199 µl of the reaction solution. After a 10-min incubation at 37 °C, the fluorescence intensity (FI) of the reaction mixture was measured using a microplate reader (SH-8000; Corona, Ibaraki, Japan) with excitation/emission wavelengths of 497/527 nm. These combined processes could be completed within 15 min.

### Detecting bile leakage on hepatic raw surfaces by fluorescence imaging using apoUnaG

1. Four wedge resections were conducted on a freshly extracted swine liver (Tokyo Shibaura Zohki, Tokyo, Japan). For two of the resections, hepatic parenchyma was dissected using a clamp-crushing method with ligation of hepatic vessels as performed during hepatectomy in humans. For the other two resections, hepatic parenchyma was sharply cut with a scalpel without sealing any of the hepatic vessels. The hepatic raw surfaces were wiped with gauze to remove any blood clots and then sprayed with a solution containing apoUnaG (3 µM). After 10 min, fluorescence images were acquired using a Maestro™ imaging system (Cri, Woburn, MA, USA) (excitation 445–490 nm; emission 515-nm-long pass). Regions of interest (5 × 5 pixels) were created to calculate the mean FI of the fluorescing spots and surrounding hepatic parenchyma.
2. In a swine laparoscopic hepatectomy model, hepatic parenchyma was divided with a vessel-sealing system under general anesthesia. After removing blood clots, apoUnaG (3 µM) was sprayed on the hepatic raw surfaces, and fluorescence images were acquired with the prototype of a fluorescence laparoscopic imaging system (Olympus, Tokyo, Japan) with excitation 465–500 nm and an emission notch filter (excitation light is blocked from 480 to 505 nm).

### Statistical analyses

Continuous data are expressed as the medians (ranges). Quantitative and categorized variables were compared using Wilcoxon’s rank-sum test and Fisher’s exact test, respectively. Correlations between UnaG FIs and the bilirubin levels measured by enzymatic methods were evaluated using Spearman’s rank correlation test.

Postoperative bile leakage was diagnosed when the TB in the drained abdominal fluid measured by the conventional enzymatic method was ≥ 3 mg/dL. The capability of UnaG-based fluorescence imaging to identify bile leakage on hepatic raw surfaces was assessed using the receiver-operating characteristics curve analysis. Statistical analysis was performed with IBM SPSS Statistics for Windows (version 21.0; IBM, Armonk, NY, USA).

## Results

### Patients’ demographics and surgical outcomes based on conventional techniques for diagnosing postoperative bile leakage

Table 1 shows the demographic backgrounds of the 23 patients in this study (22 underwent hepatectomy, 1 had a liver transplant). The preoperative serum TB levels were within the normal range (< 1.2 mg/dL) for the 22 hepatectomy patients. Pathological examination of the background liver identified normal tissue in 13 patients, chronic hepatitis in 8 patients, and liver cirrhosis in 1 patient.

**Table 1:** Patients’ demographic characteristics

Characteristic	Value
Age (y)*	66 (58–78)
Sex, male (%)	14 (61)
Preoperative liver function	
Serum total bilirubin (mg/dL)*	1.0 (0.5–10.4)
Prothrombin activity (%)*	100 (47–100)
ICG retention rate at 15 min (%)*	9.6 (3.7–20)
Child–Pugh class, A/B/C	22/0/1
Hepatitis B or C virus infection, yes (%)	7 (30)
*Preoperative diagnosis*	
Primary liver cancer	8
Colorectal liver metastasis	8
Others^†^	7
Pathological diagnosis of background liver, normal/chronic hepatitis/liver cirrhosis	14/8/1

^*^Median (range)

^†^Hilar bile duct cancer (*n* = 2); intraductal papillary neoplasm of the bile duct (1); intrahepatic calculus (1); liver cyst (1); primary sclerosing cholangitis (1, recipient of living-donor liver transplantation); donor of liver graft (1)

Surgical procedures and operative outcomes are summarized in Table 2. Bile leakage was defined as the situation in which the TB level in aspirated ascites measured by the conventional enzymatic method was ≥ 3 mg/dL. Postoperative bile leakage developed in three hepatectomy patients (13%). Because their bile leaks were all transient, no re-operation was required.

**Table 2:** Surgical outcomes

Outcome	Value
Operation time (min)*	395 (145–752)
Estimated blood loss (mL)*	605 (50–3410)
*Surgical procedure*	
Hemi-hepatectomy or larger resection^†^	6
Anatomic segmentectomy or wedge resection	15
Living-donor liver transplantation	1
Postoperative bile leak^‡^, Yes (%)	3 (13)
Postoperative complications, Clavien–Dindo classification 0–II/III	22/1^§^
Postoperative hospital stay (d)*	12 (8–84)

^*^Median (range)

^†^Including extended right hepatectomy with hepatico-jejunostomy (*n* = 1)

^‡^Total bilirubin in drained abdominal fluids measured by conventional enzymatic method was ≥ 3 mg/dL

^§^The complication of grade 3 is a case in which stent placement was necessary for bile duct stenosis after living-donor liver transplantation

### Diagnosing postoperative bile leakage based on fluorescence measurement of bilirubin levels in abdominal drainage fluids using APOUNAG

**Fig. 1:**
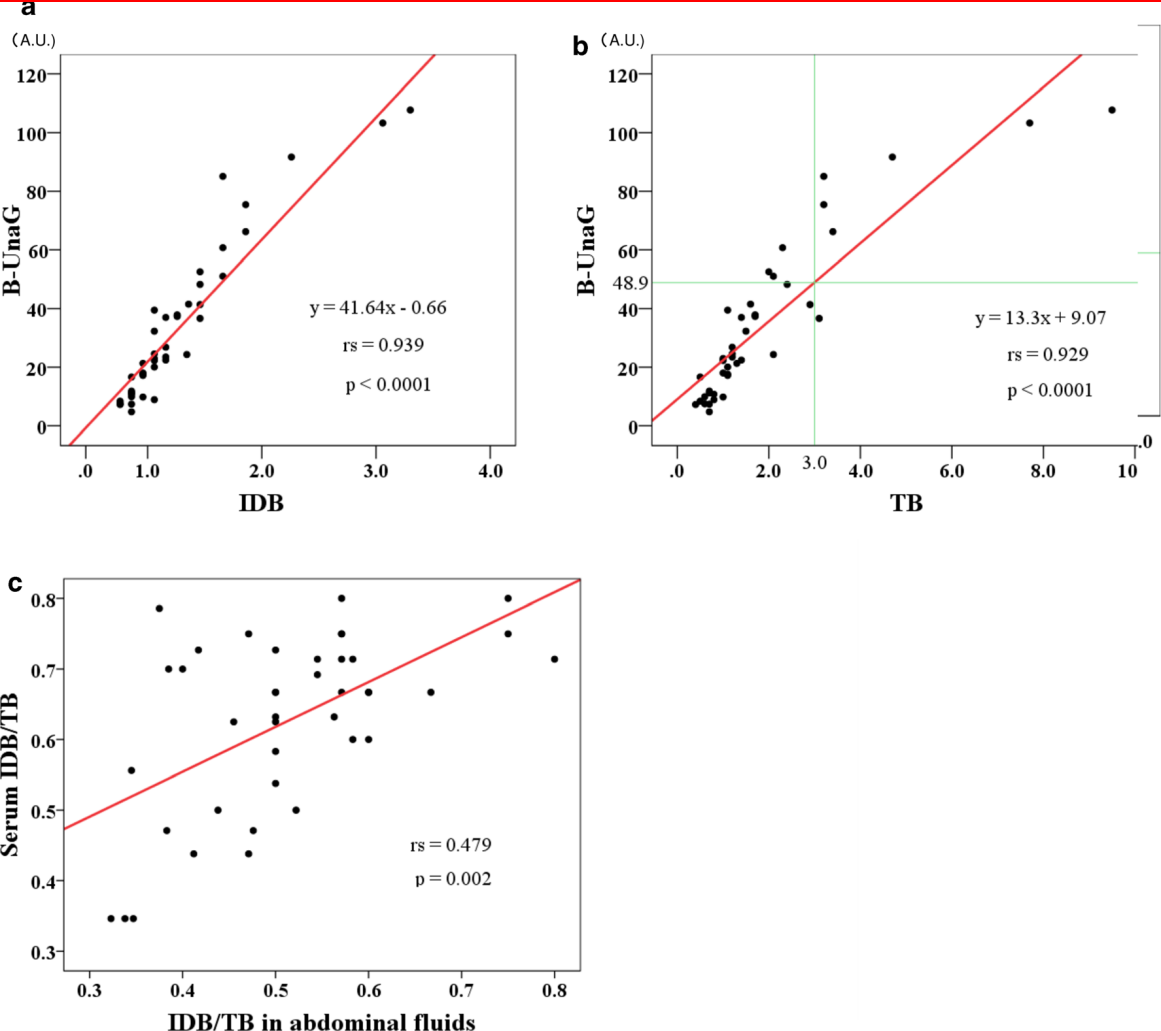
Correlations between bilirubin levels in abdominal fluids calculated using the conventional enzymatic method and those measured with UnaG. UnaG FI in drained abdominal fluids showed significant positive and linear correlations with the indirect bilirubin (IDB) level [Spearman’s rank correlation coefficient: *R*_s_ = 0.938; *P* < 0.001 (**a**)] and total bilirubin (TB) level [(*R*_s_  = 0.928, *P* < 0.001 (**b**)). The IDB/TB ratio in abdominal fluid samples ranged from 0.32 to 0.80 (median 0.50) and showed positive correlations with the serum IDB/TB ratio measured the same day (**c**)

Altogether, 42 postoperative ascites samples were collected from the 23 patients. The UnaG FIs showed significant positive and linear correlations with the IDB (Spearman’s rank correlation coefficient, rs = 0.939, *p* < 0.001) (Fig. 1a) and the TB (rs = 0.929, *p* < 0.001) (Fig. 1a) levels. The median (range) values of IDB and TB were 0.6 (0.3–3.3) mg/dL and 1.2 (0.4–9.5) mg/dL, respectively. The IDB/TB ratio in the drained ascites samples ranged from 0.32 to 0.80 (median 0.50) and showed a positive correlation with the serum IDB/TB ratio measured on the same day (Fig. 1c).

Based on the linear regression model [UnaG FI = 13.3 × *TB* + 9.07 (*R*^2^ = 0.794)] (Fig. 1b), the threshold of diagnosing postoperative bile leakage (TB ≥ 3 mg/dL in drained ascites samples) was 48.9 arbitrary units (A.U.) for UnaG FI. With this criterion, 6 of the 7 samples with TB ≥ 3 mg/dL and 3 of the 35 samples with TB < 3 mg/dL were diagnosed as indicating bile leakage. Table 3 summarizes the follow-up data of five patients (10 samples) who exhibited high values of both TB (≥ 3 mg/dL) and UnaG FI (≥ 48.9 A.U.) in the drained ascites fluid.

**Table 3:** Postoperative courses of the five patients diagnosed as having bile leakage

Pt. no	Pathology	Surgical procedure	Max. TB (mg/dL)	Max. UnaG FI (A.U.)	Drain removal (d)	Postoperative hospital stay (d)	Complication level*
1	Primary sclerosing cholangitis	Living-donor liver transplantation	9.5	107.7	150	84	IIIa
2	Liver cyst	Fenestration of the cyst	3.2	85.0	71	42	II
3	Hepatocellular carcinoma	Limited resection	4.7	91.6	43	21	II
4	Colorectal liver metastases	Right posterior sectionectomy	2.1	51.0	13	15	II
5	Colorectal liver metastases	Laparoscopic limited resection	2.0	52.5	3	8	0

*FI* fluorescence intensity, *Max.* maximum, *TB* total bilirubin, *A.U.* arbitrary units

Based on TB (≥ 3 mg/dL) and/or UnaG FI (≥ 48.9 A.U.) in the drained abdominal fluid

^*^Clavien–Dindo classification

### Fluorescence detection of bile spillage on cut surfaces of swine liver

**Fig. 2:**
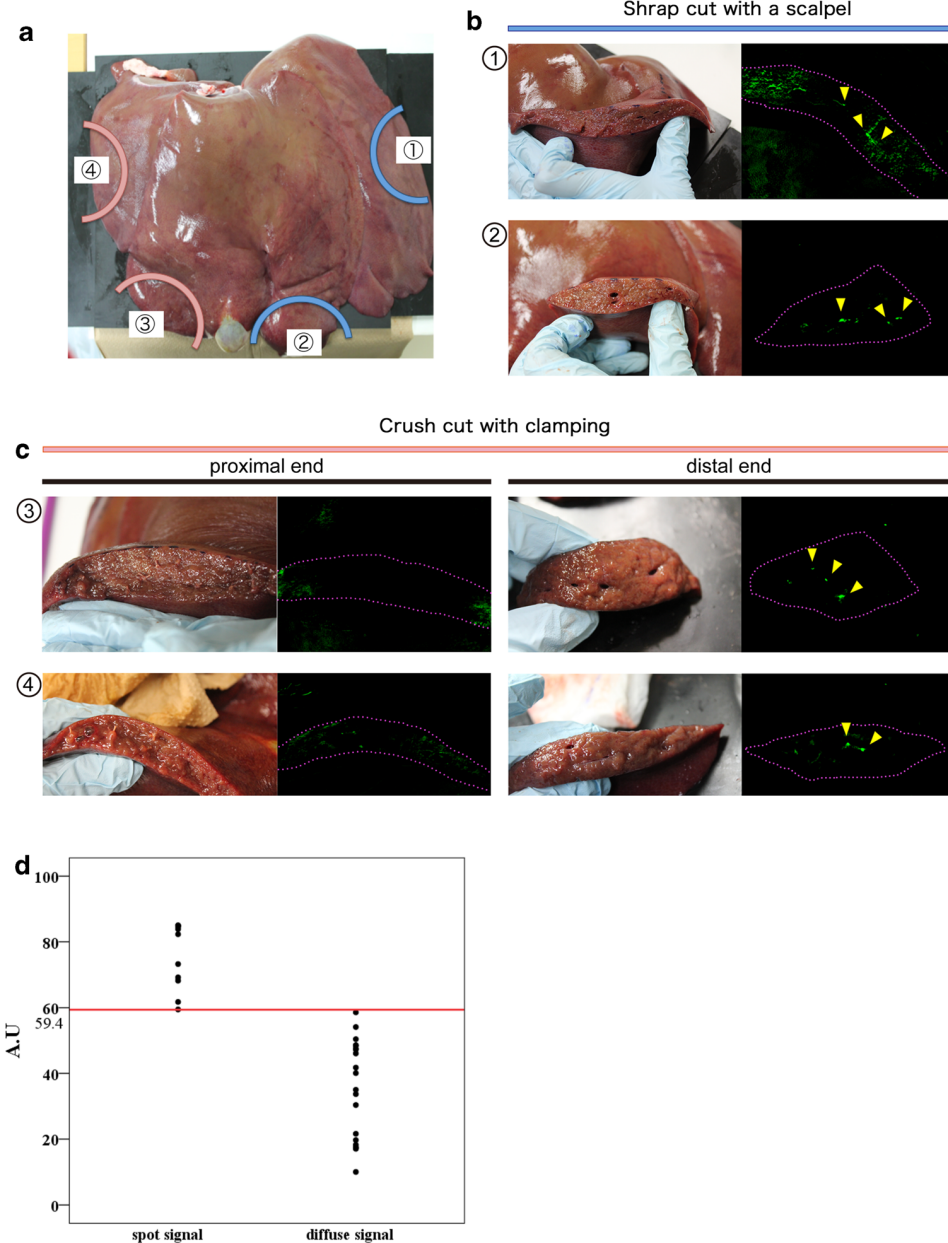
Fluorescence imaging using UnaG on hepatic raw surfaces of a fresh specimen. Hepatic transection planes were created on a fresh specimen of swine liver using a clamp-crushing method with ligation of hepatic vessels, or they were sharply cut with a scalpel without sealing any vessels (**a**). Although the fluorescence imaging visualized no fluorescent signals on the hepatic raw surfaces divided by ligation, many fluorescent spots were identified on the cut surfaces of the liver without ligation 10 min after topical administration of UnaG (**b**, **c**). Fluorescence intensity (FI) of the fluorescing spots was significantly greater than that of the diffusely fluorescent areas (**d**)

**Fig. 3:**
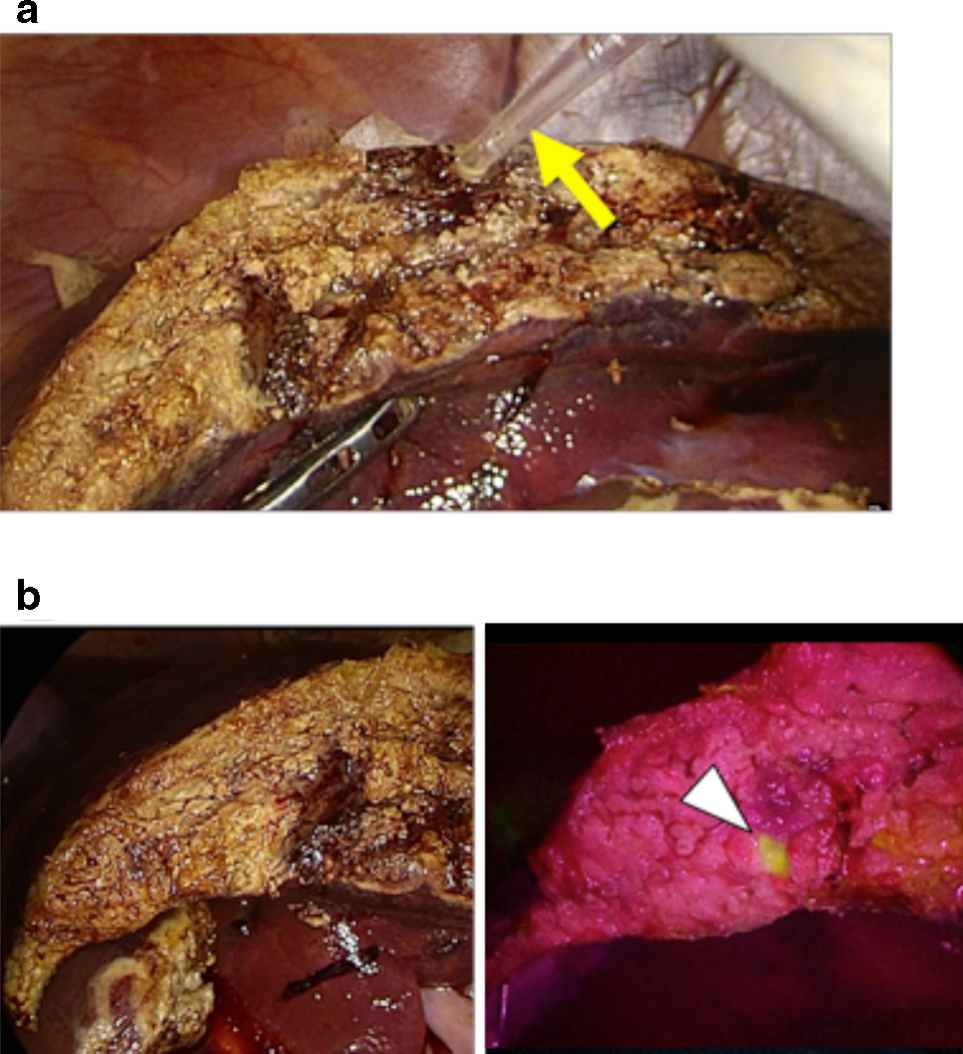
In vivo fluorescence imaging of the bile leak using UnaG in a swine model of laparoscopic hepatectomy. Following laparoscopic wedge resection of the liver, 3 µM of apoUnaG was sprayed on the hepatic raw surface using a transabdominal catheter (arrow) (**a**). Fluorescence imaging (middle and right panels) identified bile leakage as a fluorescent spot (arrowheads) on red backgrounds, beginning 1 min after apoUnaG administration. Left panel indicates white-light, full-color imaging (**b**)

A freshly extracted swine liver was used. First, two dissections were made on the liver using a scalpel (Fig. 2a, ① and ②). Fluorescence imaging with apoUnaG highlighted several fluorescent spots on the cut surfaces (Fig. 2b, ① and ②, yellow arrowheads). Although weak fluorescent signals were also observed as diffuse plaque, they were quite dim and were deemed to be bile juice that had been contaminated during the procedure. Second, two other dissections were made on the liver with ligations of hepatic vessels (Fig. 2c, ③ and ④). Although some strong fluorescent spots were found on the distal surfaces, only weak, diffuse signals were detected on the proximal ends. Our receiver-operating characteristic curve analysis revealed that there was a significant difference in the measured FI between the spots and the diffuse signals (Fig. 2d).

Next, fluorescence laparoscopy was applied to a swine hepatectomy model. Under usual white-light illumination, bile leakage spots were identifiable on the hepatic raw surfaces. However, topical administration of apoUnaG strongly emphasized the bile leakage spots within a minute (Fig. 3a, b, Supplementary Video 1).

## Discussion

The intensity of UnaG fluorescence in our ascitic fluid samples obtained from posthepatectomy patients showed positive, linear correlations with the IDB and TB levels measured by the conventional enzymatic method. When the clinically critical TB level was determined to be 3.0 mg/dL, our UnaG fluorescence-based method underdiagnosed 1 of 7 positive samples (TB ≥ 3 mg/dL) and overdiagnosed 3 of 35 negative samples (TB < 3 mg/dL). The major advantage of the in vitro diagnostic method is that it enables rapid (within 15 min) and accurate measurement of TB with a tiny drop of ascitic fluid (1 µL), with little interference from coexisting hemoglobin and lipid emulsion [18, 19]. Some portable devices that measure UnaG fluorescence are under development, which will enable surgeons and ward physicians to perform bedside diagnosis of bile leakage during morning rounds, without having to send fluid samples to the hospital laboratory to obtain bilirubin values. Such devices will also likely be adopted for bedside diagnoses of pancreatic leakage when used in combination with the fluorometric chymotrypsin probe [20, 21], which exhibits UnaG-like green fluorescence after reaction with pancreatic chymotrypsin.

The other important application of UnaG fluorescence imaging is real-time intraoperative visualization of bile leakage. Topical administration of apoUnaG in an operating field could provide surgeons with a sensitive method to detect very small amounts of bile oozing out of hepatic raw surfaces or a biliary anastomosis, neither of which is usually visible to the naked eye. Near-infrared fluorescence imaging using ICG, a water-soluble tricarbocyanine dye, has found clinical applications not only for visualizing bile duct anatomy [22] but for detecting bile leakage [23, 24]. Intravenous injection of ICG, however, renders liver parenchyma fluorescent as well, making it difficult to localize leakage sites precisely. Compared with ICG, UnaG is a native protein isolated from Japanese eel muscle and therefore should be edible. The apoUnaG protein sample used in this study was expressed in bacteria and then affinity-purified. The sample could be clinically applied after treatment with endotoxin removal resin to eliminate the endotoxins. Alternatively, recombinant apoUnaG can be produced in baculovirus-infected insect cells without concern for endotoxins. We expect that the topical administration of apoUnaG will enable surgeons to investigate bile leakage thoroughly during surgery, thereby enabling them to judge the need for prophylactic abdominal drains.

Although the physiological significance of UnaG in eel muscle remains unknown, its medical application in humans has provided several possibilities. It was already shown that apoUnaG could be used for direct in vitro measurement of IDB levels up to 60 mg/dL in newborns’ serum, thereby helping to prevent brain damage [19]. Compared with in vitro diagnostic methods, the intraoperative application is rather challenging. Considering the remarkable progress in the mass production of useful proteins in recent years, however, it will not be long before apoUnaG can be administered topically.

The major limitation of this study lies in the limited sample size with a low incidence of main outcome measures (postoperative bile leak). The reason for the inconsistency in diagnosis of postoperative bile leak between the conventional enzymatic method and the UnaG-based technique for TB measurement, which was observed only in four samples in the present series, should be analyzed based on the larger number of clinical specimens. It is also unclear whether detection of minute bile leakage in drained abdominal fluids and/or hepatic raw surfaces can lead to enhancement of postoperative outcomes. Since we have developed a portable fluorometric device enabling rapid measurement of UnaG at the bed side, further prospective studies in larger series are currently underway to validate efficacy of the present technique in the on-site management of patients after liver resection and transplantation.

## Conclusions

The UnaG-based measurement system enables rapid, accurate estimation of bilirubin levels in drained abdominal fluids after hepatectomy, which may help surgeons and ward physicians make a bedside diagnosis of a postoperative bile leak and immediately judge the need for an abdominal drain. This technique may also be applied to intraoperative identification of bile leaks by fluorescence imaging following intraabdominal topical administration of UnaG.

## Electronic supplementary material

Below is the link to the electronic supplementary material.

Supplementary file1 (MP4 33527 kb)

## Abbreviations

FI: Fluorescence intensity
ICG: Indocyanine green
IDB: Indirect bilirubin
TB: Total bilirubin
A.U.: Arbitrary units
